# SNP (A > G - rs13057211) but not GT(n) polymorphism in HMOX-1 promotor gene is associated with COVID-19 mortality

**DOI:** 10.1186/s12890-023-02785-x

**Published:** 2023-12-21

**Authors:** Kerolos Fares, Mona K. El-Deeb, Omar Elsammak, Amged Ouf, Hesham Mahmoud Sayd Saeed, Ayman Baess, Mohamed Elsammak, Eman El-Attar

**Affiliations:** 1 Department of Chemical Pathology, Medical Research Institute, Alexandria University, Alexandria, Egypt; 2 Department of Clinical Laboratory Sciences, College of Applied Medical Sciences, Al Jouf University, Sakakah, Saudi Arabia; 3 Faculty of Medicine, Alexandria University, Alexandria, Egypt; 4 Department of Biology and Biotechnology Graduate Program, School of Sciences and Engineering (SSE), The American University in Cairo (AUC), New Cairo, Egypt; 5 Department of Biotechnology, Institute of Graduate Studies and Research Alexandria University, Alexandria, Egypt; 6 Department of Chest Diseases, Faculty of Medicine, Alexandria University, Alexandria, Egypt

**Keywords:** Serum Hemoxygenase-1, Promoter GT(n) polymorphism, COVID-19, SNP, IL-6

## Abstract

**Introduction:**

COVID-19 causes severe inflammatory respiratory distress syndrome. The global pandemic caused millions of cases of morbidity and mortality worldwide. Patients may present with variable symptoms including dyspnea, fever, and GIT manifestations. The HMOX-1 gene is located on the long (q) arm of chromosome 22 at position 12.3. HMOX-1 is expressed in all mammalian tissues at basal levels and is considered as a stress response enzyme. HMOX-1 has a specific polymorphic site with variable GT(n) repeats at the promotor region. Several authors evaluated the HMOX-1 GT(n) promoter polymorphism in different inflammatory conditions. We evaluated HMOX-1 promoter polymorphism in relation to serum Hemoxygenase level and inflammatory makers (CRP, Ferritin, PCT, IL-6 and D-dimer) in patients affected by SARS-COV-2 disease.

**Subjects and methods:**

Ninety patients confirmed to be infected with COVID-19 were followed up till the study end point (recovery and discharge or death). HMOX-1 promotor GT(n) polymorphism was evaluated using Sanger sequencing. HMOX-1 enzyme serum level was measured by ELISA and the level of different inflammatory markers was assessed by available commercial kits.

**Results:**

A novel Single nucleotide polymorphism (SNP) (A > G) - rs13057211 in the GT(n) region of HMOX-1 promoter gene was found in 40 (61.5%) COVID-19 patients out of the studied 65 patients. This (A > G) SNP was associated with higher mortality rate in COVID-19 as it was detected in 27 patients (75% of the patients who succumbed to the disease) (*p* = 0.021, Odds ratio = 3.7; 95% CI:1.29–10.56). Serum IL-6 (Interleuken-6) was positively correlated the length of Hospital Stay (LOHS) and procalcitonin (PCT); (*p* = 0.014, r: 0.651 and *p* < 0.001, r:0.997) respectively while negatively correlated with levels of HMOX-1 enzyme serum level (*p* = 0.013, r: -0.61). CRP correlated positively with LOHS (*p* = 0.021, r = 0.4), PCT (*p* = 0.044, r = 0.425) and age (*p* < 0.001, r = 0.685). Higher levels of D-Dimer and PCT were observed in patients with the long repeat. There was no significant difference between patients who recovered and those who died from COVID-19 as regards HMOX-1 level and GT(n) polymorphism.

**Conclusion:**

We report a novel SNP (A > G, rs13057211) in the GT(n) region of HMOX-1 promoter gene that was associated with mortality in COVID-19 patients, however no significant difference was found in HMOX-1 serum level or HMOX-1 (GT)n repeats within the studied groups.

**Supplementary Information:**

The online version contains supplementary material available at 10.1186/s12890-023-02785-x.

## Introduction

Coronaviruses are enveloped Ribonucleic Acid (RNA) viruses belonging to the Coronaviridae family named after the “crown-like spikes” present on their surface [1]. The coronavirus disease 2019 (COVID-19) was first described in Wuhan, China, in December 2019 and ever since, the outbreak has rapidly spread across the world. COVID-19 is caused by a novel beta-coronavirus named severe acute respiratory syndrome coronavirus 2 (SARS-CoV-2) [2]. Most of COVID-19 cases (about 85%) develop mild symptoms while 5% of infected patients have severe disease characterized by acute respiratory distress syndrome (ARDS) and multi-organ damage [3]. The mortality of Intensive Care Unit (ICU) patients is mainly due to ARDS which accounts for 60% of COVID-19 deaths, especially in the elderly [4]. COVID-19 disease caused severe global pandemic with severe morbidity and mortality. Till the time of writing this manuscript, the number of deaths caused by COVID-19 reached 6.8 million deaths globally [5]. The striking difference between SARS-CoV-2 and previous Corona virus strains is the high virulence of SARS-CoV-2 strain with subsequent severe clinical manifestations [6].

There is increasing evidence that cytokine storm syndrome may play a critical role in severe COVID-19. The balance between pro-inflammatory and anti-inflammatory cytokines is regulated by several mechanisms that normally limit the inflammatory process. Loss of one or more of these regulatory processes may lead to the overstimulation of the immune system [7]. One of the virulence factors of SARS-CoV-2 is its spike proteins which affect cells by dysregulation of multiple inflammation regulatory cytokines as Tumor Necrosis Factor- α (TNF-α), Interleukin-1β (IL-1β) and Nuclear Factor Kappa B (NF-κB) leading to marked pro-inflammatory response. SARS-CoV-2 also induces Angiotensin Converting Enzyme 2 (ACE2) and massive release of cytokines [8].

Patients usually present with fever, dry cough, shortness of breath and diarrhea. SARS-CoV-2 can also cause multi-organ damage with extra-pulmonary manifestations and possible progression to severe disease and death [9]. Current therapeutic approach focuses on targeting COVID-19-induced cytokine storm syndrome to reduce SARS-CoV-2 mortality and limit ICU overload [10].

Hemoxygenase 1 (HMOX-1) is an enzyme that catalyzes the first step of the oxidative degradation of the heme group. Hemoxygenase is a rate-limiting enzyme in a reaction that releases several by-products, including carbon monoxide (CO), biliverdin (which is reduced to bilirubin) and free iron [11]. The latter can be effectively controlled by ferritin via sequestration and ferroxidase activity. These homeostatic adjustments have been shown to be effective in protecting the endothelium from the deleterious effects of heme and iron free radicals. Lack of free radical control in an iron-rich environment may result in extensive endothelial damage in humans [12]. On the other hand, Carbon monoxide has multiple important protective roles; including a vasodilator effect [13], neuroprotective role [14] and more importantly an anti-inflammatory role [15].

HMOX-1 is composed of 288 amino acid residues with an active site located between the first two alpha helixes. HMOX-1 expression can be induced either by substrate (heme) or by other physical and chemical stimuli, including metalloporphyrins, oxidative stress and cytokines [16]. The *HMOX-1* gene is located on the long (q) arm of chromosome 22 at position 12.3. HMOX-1 is expressed in all mammalian tissues at basal levels. HMOX-1 expression levels can significantly increase in cells or tissues where red blood cells or hemoglobin are degraded by macrophages, such as the spleen, liver, bone marrow, and kidney [17].

The possible relation between genetic polymorphism in Hemoxygenase enzyme promoter gene, the severity of COVID-19 disease and patient outcome was not previously investigated. The current prospective study aimed at the evaluation of *HMOX-1* promoter gene polymorphisms - GT(n) and potential SNPs - and HMOX-1 enzyme serum level in relation to prognosis and outcome of COVID-19 disease in hospitalized patients. The study also investigated whether there is a correlation between *HMOX-1* promoter gene polymorphism, serum HMOX-1 enzyme level and several inflammatory and clinical markers in COVID-19 patients.

## Subjects and methods

The study comprised 90 PCR (polymerase chain reaction) positive hospitalized COVID-19 patients. Patients age was 68 ± 15 years (mean ± SD). The patient’s cohort comprised of 49 female patients (54%) and 41 male patients (46%). Exclusion criteria included malignancy, chronic lung diseases, end stage renal disease (stage 4 or 5), patients taking immunosuppressive medications or those who had history of chemotherapy. Radiological examination with CT-Chest and a battery of laboratory tests were done for all patients included in the study. Samples were collected during the first day of admission and the following tests were performed; CBC (complete blood count), serum Alanine Transaminase (ALT), Aspartate Transaminase (AST), Urea, Creatinine, Lactate Dehydrogenase (LDH), plasma D-dimer, Prothrombin Time, and Activated Partial Thromboplastin Time. The following inflammatory markers: C-reactive protein, IL-6 and Ferritin were also measured.

Serum HMOX-1 enzyme level was measured using commercially available Enzyme Linked Immunosorbent Assay (ELISA) kit (Cloud-Clone corporation, Catalogue No.E-00733hu, Houston, USA).

Determination of the *HMOX-1* GT(n) repeats was done as described elsewhere [18]. Briefly, DNA was extracted from peripheral blood using Qiagen DNA extraction kit (QI Amp DNA blood Mini kit, Catalogue No./ ID: 51104, Germany). The extracted DNA was amplified using polymerase chain reaction. A forward primer 5’ (AGAGCCTGCAGCTTCTCAGA) 3’ and reverse primer 5’ (ACAAAGTCTGGCCATAGGAC) 3’ were used [19].

PCR conditions included initial denaturation step for 5 min at 95 °C followed by 30 cycles of denaturation for 30 s at 95 °C, annealing for 15 s at 58 °C and extension at 72 °C for 45 s, a final step of extension step for 5 min at 72 °C was performed. The amplicons were purified using Geneaid Gel/PCR DNA fragments purification kit (Catalogue No. DF100/300, Taiwan). The purified amplicon was sequenced using Sanger sequencing technique as described elsewhere [20]. The results were analyzed using Bio-Edit software. Results were analyzed by two independent researchers to confirm the GT repeats length and the presence of any SNP (Single Nucleotide Polymorphism). Statistical analysis of results was carried out using SPSS statistical package version 24 (SPSS, Inc., Chicago, IL, USA). The chi-square test was used for comparisons of the categorical variables between different groups. The Fisher exact test was also applied when > 20% of the variables had an expected count of < 5.

*p* value of < 0.05 was considered to indicate statistical significance. The Kolmogorov-Smirnov test for normality was used to evaluate the degree of deviation from a normal distribution across all the variables. A Student T-test and Mann-Whitney test were applied to normally and abnormally distributed quantitative variables, respectively.

## Results

**Fig. 1:**
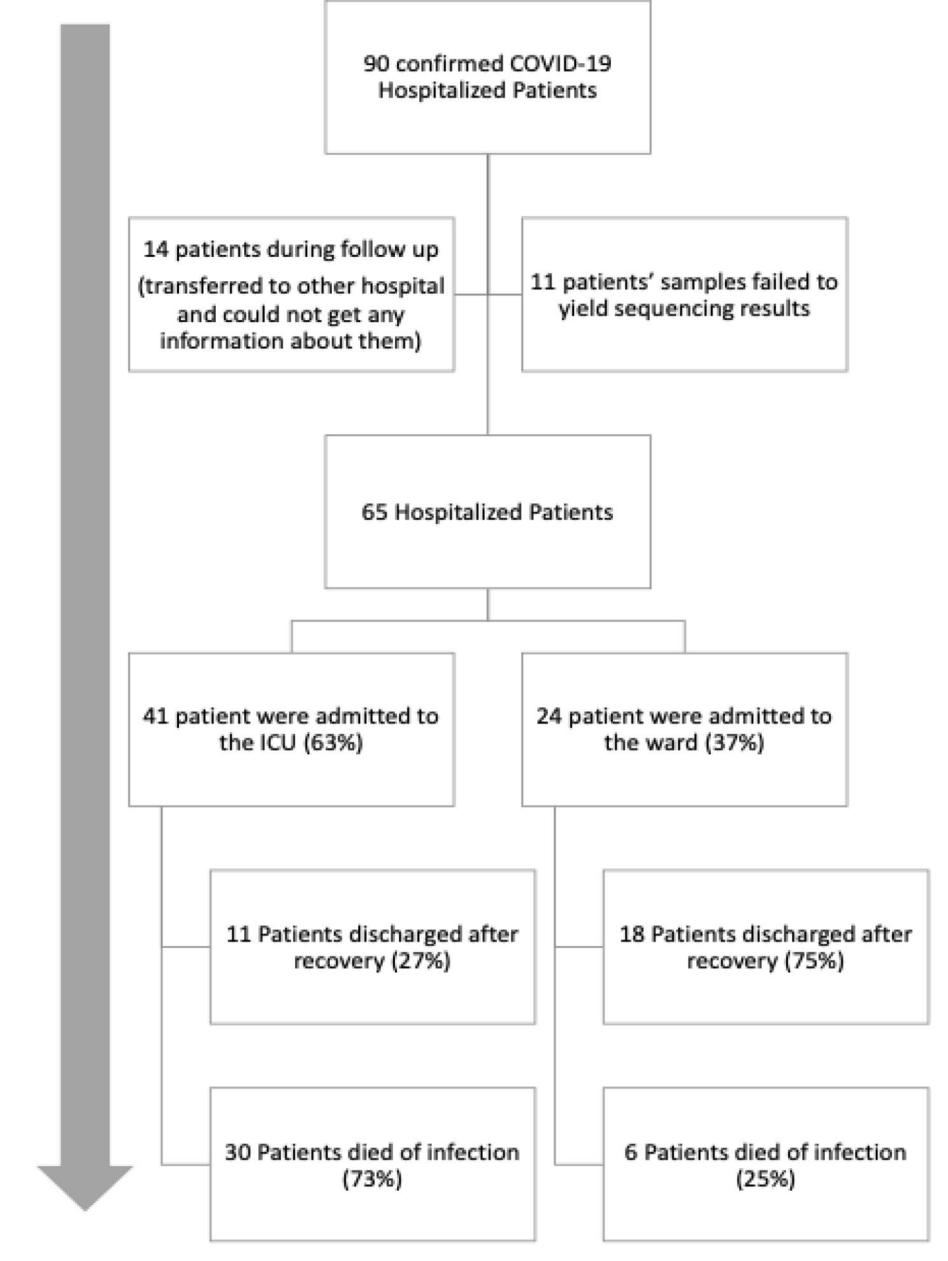
Funnel diagram illustrating the patient cohort along the course of the study

The current work is a prospective study that followed 90 hospitalized Egyptian patients affected with COVID-19 (PCR confirmed). Twenty-Five patients were lost during follow up. Patients were stratified into two groups; patients who survived and those who died during COVID-19 disease. Figure 1 describes sample size, patient selection, and classification according to the course of the disease and outcome. Twenty-nine patients (44.6%) were discharged after resolution of the disease while 36 patients (55.4%) died.

Demographic and clinical data of both groups are summarized in Table 1. The laboratory data are illustrated in Table 2. There was a significant difference between studied groups as regard CRP (*p* = 0.048) and PCT (*p* = 0.015), where higher levels were found in the deceased group. The two groups showed no significant difference in IL-6 and D-dimer levels. Serum level of HMOX-1 was not significantly different between survivors and those who died as a result of COVID-19 (*p* = 0.276).

**Table 1:** Demographic and clinical data of studied COVID-19 patients

		Died n = 36		Discharged n = 29		*p*-Value
		n (%)		n (%)		
Gender	Female	20 (56)		14 (48)		0.559 ^¶^
	Male	16 (44)		15 (52)		
		Mean	SD	Mean	SD	
Age (yrs)		61.45	± 14.14	59.83	± 13.2	0.633 ^§^
Pulse (bpm)		89.86	± 11.8	83.75	± 20.9	0.327 ^§^
RR (cpm)		22	± 2.66	22.63	± 4.1	0.632 ^§^
		Median	Range	Median	Range	
LOHS (days)		7.8	2–30	7.67	1–27	0.416 ^†^
Temperature (°C)		37.5	37–38.2	37	36.5–38	**0.028** ^†^
SO2 (%)		95	67–99	95	67–100	0.748 ^†^
pH		7.36	7.3–7.5	7.5	7.4–7.53	**0.01** ^†^
HCO3 (mEq/L)		24	18–59	26	22.8–29.6	0.651 ^†^
PaO2 (mmHg)		76	45–172	95	72–172	0.169 ^†^
PaCO2 (mmHg)		34	16–55	33	16.4–38	0.91 ^†^
SBP		140	100–160	140	110–170	0.324 ^†^
		n (%)		n (%)		
Setting of admission	ICU	30 (83)		11 (38)		**< 0.001** ^¶^
	Ward	6 (17)		18 (62)		
Type of Ventilation	MV	25 (69)		6 (21)		**< 0.001** ^¶^
	O2	11(31)		23 (79)		

Demographic data of the patients included in the study. Normally distributed variables were expressed as Mean ± SD while abnormally distributed variables were expressed as Median (Range)

n = number, LOHS: Length of hospital stay, RR: Respiratory rate, SO2: Oxygen saturation, SBP: Systolic Blood Pressure, SOA: setting of admission, TOV: Type of Ventilation, MV: Mechanical Ventilation, O2: Simple Oxygen

Significant *p*-value: less than 0.05

^§^ : t-test

^†^ : mann-whitney test

^¶^ : Pearson

**Table 2:** Biochemical data of studied COVID-19 patients

	Died		Discharged		*p*-value
	Median	Range	Median	Range	*p*-Value
Urea (mg/dL)	46	19–175	35	9–220	0.409 ^†^
Creatinine (mg/dL)	1.0	0.4–4.2	0.9	0.5–3.32	0.849 ^†^
AST (U/L)	35	18–89	34	18–107	0.681 ^†^
ALT (U/L)	37	15–88	53.5	14–223	0.104 ^†^
TBIL (mg/dl)	0.6	0.3–0.9	0.8	0.2–0.9	0.762 ^†^
D-dimer (ng/ml)	540	190–7170	420	190–2440	0.282 ^†^
Ferritin (ng/ml)	631	110–1650	610	40–1650	0.288 ^†^
LDH (U/L)	502.5	157–2805	298	202–990	0.428 ^†^
RBS (mg/dL)	194	74–450	180	174–200	0.727 ^†^
CRP (mg/L)	108	3.5–510	113	4.4–257	**0.048** ^‡^
IL-6 (pg/ml)	104	12.3–5000	33	1.5–185	0.171 ^†^
PCT (ng/ml)	0.225	0.01–38.47	0.12	0.01–0.6	**0.015** ^‡^
	**Mean**	**SD**	**Mean**	**SD**	*p*-Value
HMOX-1 (µg/ml)	54.12	± 30	61.25	± 19.8	0.276 ^§^

Biochemical data of the patients included in the study. Normally distributed variables were expressed as Mean ± SD while abnormally distributed variables were expressed as Median (Range)

AST: Aspartate Transaminase, ALT: Alanine Transaminase, TBIL: Total bilirubin, LDH: Lactate Dehydrogenase, RBS: Random Blood Sugar, CRP: C-Reactive Protein, PCT: Procalcitonin, HMOX-1: Hemoxygenase enzyme serum level

Significant *p*-value: less than 0.05

^§^ : t-test

^†^ : mann-whitney test

^¶^ : Pearson

^‡^: Levene’s test

**Fig. 2:**
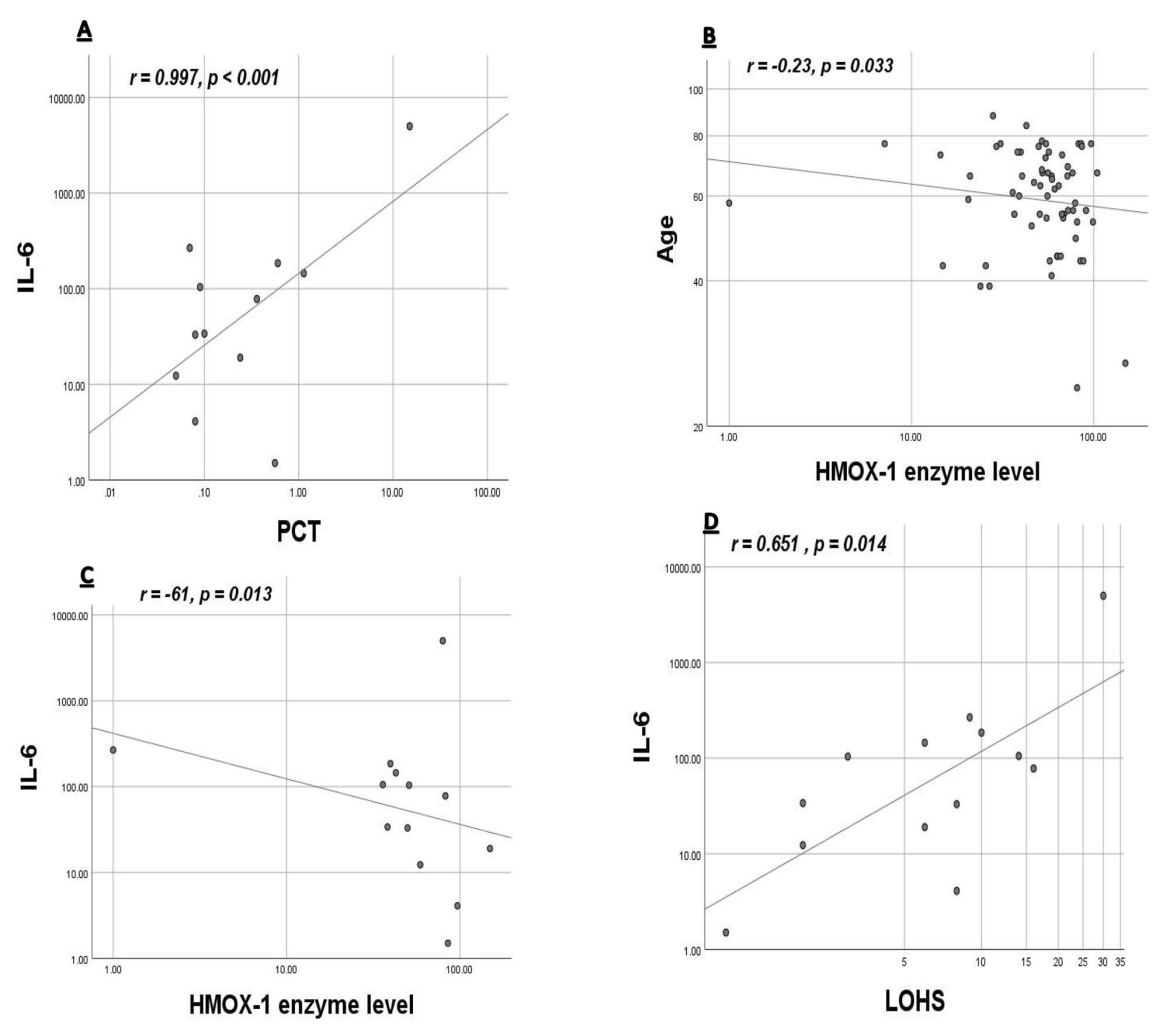
Correlations between different parameters evaluated in the studied population A- positive correlation between Interleukin- 6 (IL-6) and Procalcitonin (PCT), B- negative correlation between Age and Hemoxygenase (HMOX-1) enzyme level, C- negative correlation between IL-6 and HMOX-1 enzyme level and D- positive correlation between IL-6 and length of hospital stay (LOHS) R (correlation coefficient) and *p* values are shown. *P* < 0.05: Significant correlation

When all cases were analyzed collectively, IL-6 correlated positively with LOHS (*p* = 0.014, r = 0.651) and PCT (*p* < 0.001, r = 0.997). Similarly, CRP correlated positively with LOHS (*p* = 0.021, r = 0.4), PCT (*p* = 0.044, r = 0.425) and age (*p* < 0.001, r = 0.685). HMOX-1 enzyme serum level showed a negative correlation with IL-6 (*p* = 0.013, r= -0.61) and age (*p* = 0.033, r = -0.23). Figure 2 summarizes the correlations between different parameters evaluated in the current study.

**Fig. 3:**
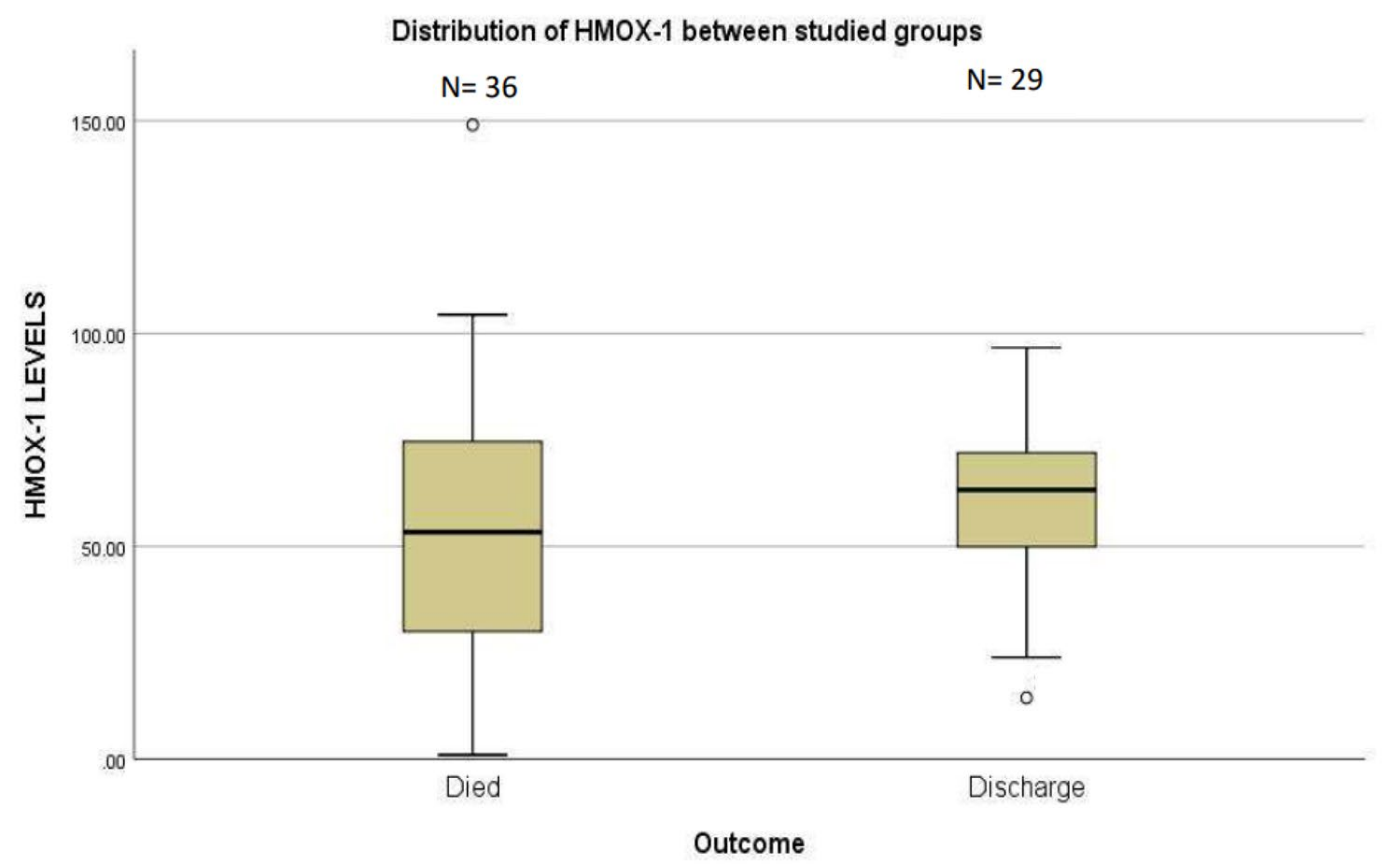
Illustrate serum HMOX-1 level in survivors and those who died during COVID-19. Boxplots illustrate serum HMOX-1 (pg/mL) in patients passes away and those who discharged post COVID-19. Number of patients included in each group was 36 Died and 29 discharged COVID-19 diseases. No significant difference was noted between the two groups regarding HMOX-1 level. *p* value = 0.276. The boxplots represent the interquartile range from the 25th to the 75th percentiles. The whiskers below and above the boxes represent the minimum and maximum values, respectively. The line across each box represents the median value. N = number of patients included in each group. O: Outliers (values larger than the upper quartile plus 1.5 times the interquartile range)

Although the level of serum HMOX-1 was higher in survivors than those who succumbed to COVID-19 disease, this difference was not statistically significant (*p* = 0.276). Figure 3 illustrates the level of serum HMOX-1 in the two groups.

Patients were further classified according to the number of GT repeats as those having long (≥ 22) or Short (< 22) repeats. Twenty-two patients (34%) had short GT repeat while 43 (66%) had long GT repeats. Based on this classification, patients having the long repeats showed significantly higher levels of PCT (*p* = 0.043) and D-Dimer (*p* = 0.048) than those having the short repeats. However, there was no significant difference between the two groups regarding the levels of HMOX-1 (*p* = 0.161). Similarly, there was no significant difference between survivors and those who died during COVID-19 disease regarding the length of GT(n) repeats (*p* = 0.667). (Table 3)

**Table 3:** GT repeat length in the studied COVID-19 patients

		GT repeat length		*p*-value
		Short	Long	
		n (%)	n (%)	
Outcome	Died	13 (59)	23 (53)	0.667 ^¶^
	Discharge	9 (41)	20 (47)	
		Median (Range)	Median (Range)	
PCT (ng/ml)		0.09 (0.01–0.42)	0.3 (0.1–38)	**0.043** ^†^
LDH (U/L)		659 (487–2805)	307 (157–990)	**0.011** ^†^
		Mean ± SD	Mean ± SD	
D-dimer (ng/ml)		437 ± 290	1584 ± 1943	**0.048** ^§^
CRP (mg/L)		197 ± 175	103 ± 88	**0.003** ^‡^
HMOX-1 (µg/ml)		55.8 ± 21.3	60.5 ± 27.8	0.161 ^§^

Table 3 summarizes the percentage of the short and long GT repeats alleles in survivors and patients who died as a result of COVID-19 disease. The concentrations of different markers evaluated in the study were stratified according to the GT repeat length whether short or long repeats. Normally distributed variables were expressed as Mean ± SD while abnormally distributed variables were expressed as Median (Range)

CRP: C-Reactive protein, PCT: procalcitonin, LDH: Lactate dehydrogenase and HMOX-1: Hemoxygenase enzyme

§ : t-test

† : Mann-Whitney test

¶ : Pearson

‡: Levene’s test

Significant *p*-value: less than 0.05

**Fig. 4:**
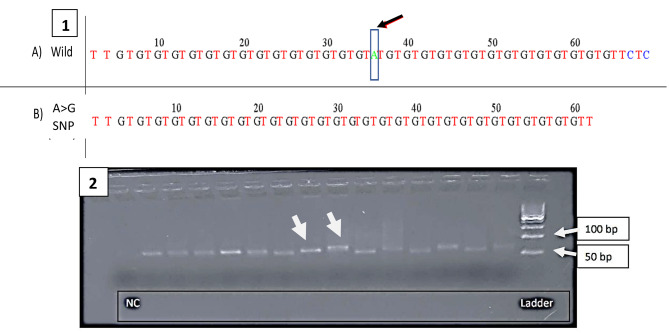
Sequence results and agarose gel electrophoresis of 2 representative samples from the studies cohort The upper panel (1) shows the sequence of the wild type A (Genbank S58267) while (B) shows the the (A > G) SNP (rs13057211) The lower panel (2) shows agarose gel electrophoresis of the amplified segment (~ 80 bp) of *HMOX-1* promoter gene in some samples.The amplicon (Thick arrow pointing at the 80 Bp fragment) was subsequently purified and sequenced. The two thin arrows show the ladder marker fragments of 50 and 100 Bp respectively NC: negative control Ladder: DNA ladder bp: base pairs

Interestingly, when the sequence of each case included in the study was examined thoroughly, a (A > G) SNP (rs13057211) in the GT(n) region of *HMOX-1* promoter gene was found. (Fig. 4)

**Fig. 5:**
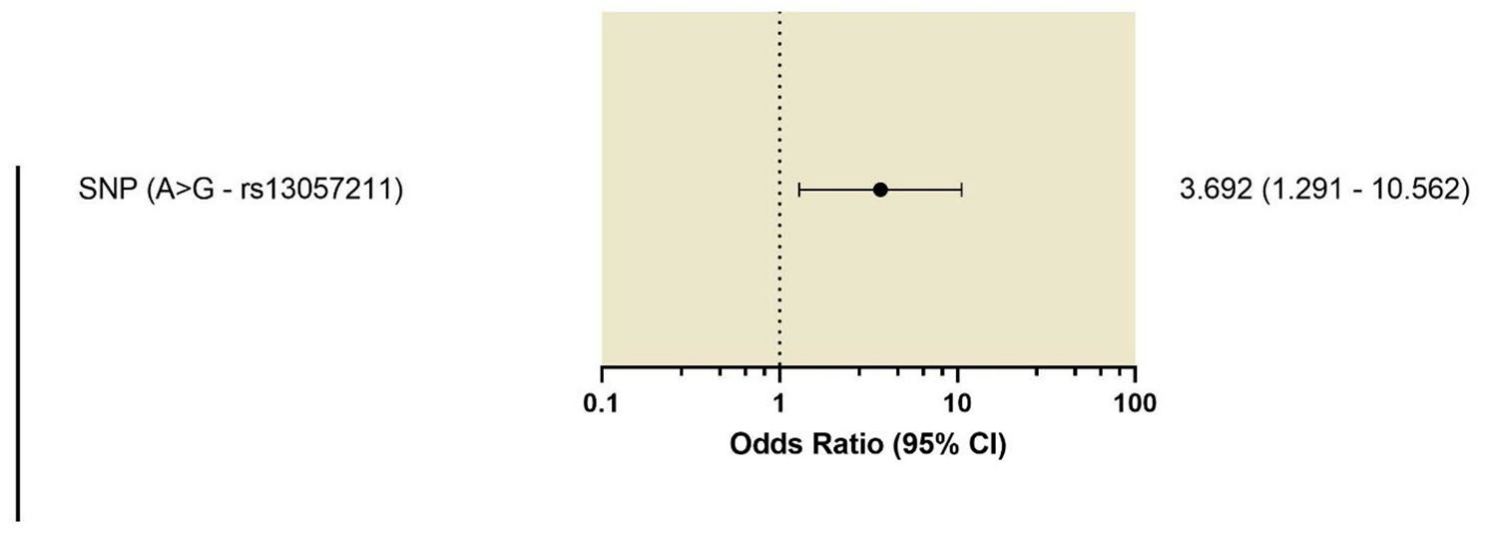
Risk of mortality in the patients harboring the SNP (A > G- rs13057211) There was a statistically significant increased risk of mortality in the presence of SNP (A > G- rs13057211) Calculated Odds Ratio was 3.7 with a confidence interval of (95% CI: 1.29–10.56)

This (A > G) SNP (rs13057211) was found in 40 patients out of 65 included in the study (62%). Most importantly, there was a significant association between the (A > G) SNP (rs13057211) and mortality (*p* = 0.021). When this (A > G) SNP was examined in patients who succumbed to COVID-19, twenty-seven patients (75%) out of all 36 deceased patients harbored the (A > G) SNP (rs13057211). An overall mortality rate of 41.5% was observed (27 out of 65) and this has an odds ratio of 3.7; 95% CI (1.29–10.56). (Table 4) (Fig. 5).

**Table 4:** (A > G) SNP (rs13057211) in patients who survived and those who succumbed to COVID-19 disease

Outcome	(A > G) SNP (rs13057211)		Total	*p*-value	OR (95% CI)
	Present	Absent			
Died (n)	27	9	36	0.021*	3.7 (1.29–10.56)
Discharged (n)	13	16	29		
Total	40	25	65		

Table 4, shows the frequency and the results of the cross tabulation of the (A > G) SNP (rs13057211) and the outcome in the studied groups. There was a clear significant difference regarding the distribution of (A > G) SNP (rs13057211) in the two studied groups (*P* < 0.05). Chi square test (Exact Fisher test) was used

When patient’s cohort was stratified according to the presence or absence of (A > G) SNP (rs13057211), no statistically significant association could be found with other clinicopathological variables (data not shown).

## Discussion

COVID-19 may present with severe acute respiratory syndrome with microvascular thrombosis, vascular injury, and life-threatening respiratory distress syndrome [3]. The underlying mechanism of COVID-19 manifestations are poorly understood. Sepsis, severe cytokine storm and immune dysregulation have been suggested as possible mechanisms [21]. Sepsis is characterized by a systemic inflammatory response syndrome (SIRS) with overexpression of proinflammatory cytokines and may lead to lethal multi-organ failure [22]. In addition, the inflammatory process is increasingly recognized as an important mechanism regulating thrombus formation and thrombolysis [23]. However, the variable outcome of different patients affected with COVID-19 disease is still puzzling.

Hemoxygenase enzyme plays a key role in heme metabolism. Oxidation of heme results in the production of biliverdin, free iron and carbon monoxide. The products of heme catabolism are under strict control to avoid tissue injury from free iron and carbon monoxide [24]. Hemoxygenase is induced in response to stress and plays an important role in controlling the inflammatory process and tissue damage that may occur in various diseases [25]. HMOX-1 was found to have an anti-inflammatory properties and protective effect against lipopolysaccharide induced lung injury [26].

Studies have shown its protective effect on various organs as liver, heart, and lung from various insults [25, 27, 28]. Deficiency of Hemoxygenase may result in uncontrolled inflammation, coagulopathies [29], vascular injury and hemolytic anemia [27, 30].

*HMOX-1* has a specific polymorphic site at the promoter region. Polymorphisms in the human *HMOX-1* promoter are characterized by the presence of several GT repeats. Several common genetic variations including GT(n) dinucleotide repeats and single nucleotide polymorphisms (SNPs) within the *HMOX-1* promoter region were reported previously [31]. Genetic variations in the promoter gene may modulate the level of promoter transcriptional activity and hence HMOX-1 serum level and may be responsible for the variability in executing the HMOX-1- stress response effects [32].

Several authors reported the association between decreased repeat number and the decrease in HMOX-1 expression and/or HMOX-1 activity with a subsequent poor outcome in many conditions [33, 34]. However, the association between number of repeats and the outcome of several diseases have shown discrepant results. Some authors illustrated an association between short alleles and poor outcome [35, 36], while others found a more favorable outcome with the short allele [37, 38], and some reported lack of association [39, 40].

The current study showed GT(n) repeat length that typically varied from 12 to 30 repeats with a trimodal distribution. Most common repeats were 16, 23 and 29. Our results of trimodal distribution are similar to those obtained in studies that evaluated cohorts of African ancestry [31]. *HMOX-1* promoter gene GT repeats were classified according to number of repeats into short repeats (< 22) or long repeats (≥ 23 repeats).

Different classifications of *HMOX-1* promoter gene repeats in various diseases have been reported; some describe them as short and long with different cut offs (22, 26 or 27) [35, 41, 42]. Other authors reported the repeats as three groups short, medium, and long with cut offs (23 or 25, 28 or 29 and 29 or 30) [34, 43]. GT(n) microsatellite polymorphism was studied in various settings regarding its possible effect on severity and outcome of infections, malignancies, and neonatal disorders [31, 44]. Discrepant results were noted between different authors. Shue et al. illustrated that longer GT- promoter repeats are associated with lower risk of ARDS [45] while Yasuda et al. found a higher risk for pneumonia patients with shorter alleles [46]. Conversely Hausmann et al. and Vázquez-Armenta et al. found no difference in the distribution of the alleles among the studied groups as regards inflammation and infection respectively which is concordant with our findings [37, 47].

The length of GT repeats has not been investigated in relation to COVID-19 outcome previously. In the current study there was no significant difference in the length of the repeats between survivors and those who succumbed to COVID-19 disease. The lack of association between Hemoxygenase promoter gene GT repeats polymorphism in our studied groups of patients may be explained by an ethnic difference in the Egyptian population. Collectively the evidence of the presence of an association between *HMOX-1* GT(n) promoter gene polymorphism and infectious disease is weak as shown by the systematic review of Hamilton et al. [31].

The current study reports a novel SNP, (A > G) rs13057211, in 40 of the COVID-19 patients (62%). Patients harboring this (A > G) SNP had a poor outcome compared to patients who did not have this (A > G) SNP (*p* = 0.021). The odds ratio of mortality of patients harboring this (A > G) SNP was 3.7; 95% CI (1.29–10.56) indicating a high probability of mortality in those patients. To the best of our knowledge, this polymorphism has not been reported in previous literature.

No difference was found between COVID-19 patients who survived the infection and those who passed away regarding serum Hemoxygenase level. Although HMOX-1 is a stress response enzyme and plays a crucial role in protection against heme induced cytotoxicity through the degradation of heme and scavenging iron generated free radicals, HMOX-1 level did not differ with the outcome of COVID-19. This could be attributed to the multiplexity of its inducers and regulators. The disease severity and the high mortality rate in our cohort could be another reason for the lack of difference in HMOX-1 enzyme levels between those who died and the survivors.

Discrepant results as regards HMOX-1 were recently reported. A recent study by Chen et al. found that HMOX-1 enzyme level does not differ as regard in-hospital mortality which is concurrent with our findings [48]. Conversely, one study showed difference in the level of HMOX-1 mRNA expression between critically ill patients and control groups which is discordant to our findings [49]. This difference may be attributed to the fact that they compared HMOX-1 mRNA level (not the serum level of HMOX-1) between heathy volunteers and severely ill COVID-19 patients. Furthermore, the lower mortality rate in their study could be the reason for the difference in the findings. Patients’ clinical condition was not generally poor as they had low mortality rate in the cohort about 19% while our study had a mortality rate of 55%.

The current study detected a significant negative correlation between the HMOX-1 level and age. The decline in the availability of Nicotinamide Adenine Dinucleotide NAD caused by aging [50], together with the ability of SARS-COV-2 to further reduce the availability of NAD + could potentially downregulate the activation of Sirtuins (SIRT 1–7) which are a family of Nicotinamide Adenine Dinucleotide (NAD+) dependent deacetylases that influence inflammatory and redox pathways [51]. One of which is Nuclear factor erythroid 2-related factor 2 (NRF2) activation which is an important regulator of HMOX-1 enzyme expression [52]. Altogether, these factors could be the reasons for the observed negative correlation between age and HMOX-1 level and may explain the unfavorable prognosis of COVID-19 that is observed in elderly patients [53].

Zhang et al. found that viral Non-structural protein-14 (NSP14) could impair the activation of NrF2/HMOX-1 pathway thus causing downregulation of HMOX-1 despite the oxidative stress caused by age [54]. This further illustrates the capacity of SARS-CoV-2 to block host defense mechanisms.

The elevated serum IL-6 detected in the current study may represent an immunological disturbance in COVID-19 disease with marked increase in cytokine production and augmented immune response [55]. A significant negative correlation between HMOX-1 levels and IL-6 was found when all our subjects were evaluated regardless of the outcome. This can be explained by the paradoxical relationship between IL-6 and HMOX-1 enzyme, as current evidence suggests that HMOX-1 is involved in the resolution of inflammation by modulating apoptotic cell death or cytokine expression [56].

Animal model study of *HMOX-1* gene transfer demonstrated that the transfer of *HMOX-1* complementary Deoxyribonucleic Acid (cDNA) resulted in suppression of pathological intrapulmonary changes; enhanced survival of animals; and a decrease of inflammatory cells in the lung [57].

Moreover, SARS-COV-2 antigens through the interaction with ACE2 may over activate a pro-inflammatory pathway via nuclear pyrin domain-containing protein 3 (NLRP3) inflammasome [58, 59].

NLRP3 activation leads to the release of inflammatory cytokines such as IL-6 and TNF-a that play a key role in the cytokine storm [60, 61]. Conversely SARS-COV-2 Open Reading Frame 3a (ORF3a) protein was found to bind and disrupt HMOX-1 [62]. These factors in combination may explain the negative correlation found in this study between serum HMOX-1 and IL-6.

The current study showed significant positive correlation between IL-6 and PCT and between CRP and PCT. These inflammatory markers also correlated positively with the LOHS. As would be expected; the inflammatory markers (CRP and PCT) showed higher levels in those who died of the disease. This positive correlation implies a higher disease burden and inflammatory response that necessitates longer hospital admission and the possible development of more complications including multi-organ failure and ARDS. Our finding mirrors the findings of other authors who evaluated PCT and IL-6 in COVID-19 patients [63].

We found a significantly higher level of PCT in patients with longer (≥ 23) GT(n) repeats which may point to the protective effect of the shorter repeats against sepsis and the increased risk of sepsis with the longer repeats which is concordant with previous studies [37, 64]. Interestingly the current study showed higher level of D-dimer in patients with long repeats (≥ 23 repeats) which can indicate the potential pro-thrombotic effect of long repeats as was previously described by Mustafa et al. [29].

In conclusion, the current study found no significant difference in the distribution of long and short GT(n) repeats in *HMOX-1* promoter gene among patients who survived COVID-19 disease and those who succumbed to death. However, we report a novel finding that is the detection of (A > G - rs13057211) SNP in the *HMOX-1* promoter gene that is associated with higher risk of mortality in patients affected with COVID-19 disease. To our knowledge no previous studies evaluated this (A > G - rs13057211) SNP before. This finding may be helpful in patients’ prognostication and warrants further studies to confirm this association in other ethnic populations affected with COVID-19 disease.

Future work may involve, examining the possible relationship between the detected **(**A > G - rs13057211) SNP and the severity of long-term complications in survivors of COVID-19 disease. Furthermore, the investigation of possible association between **(**A > G - rs13057211) SNP and the outcome of other inflammatory disorders represents an interesting research idea.

### Limitations of the study

The current study was performed on a relatively small number of patients; thus, bigger studies involving patients in different age groups are needed to check the consistency of the current findings.

### Electronic supplementary material

Below is the link to the electronic supplementary material.

Supplementary Material 1

Supplementary Material 2

Supplementary Material 3

## Data Availability

The datasets generated during the current study are available in the Bioproject: repository, Accession: PRJNA1015194. Link: https://www.ncbi.nlm.nih.gov/bioproject/1015194. GenBank accession numbers: OR541344-OR541442.
